# Effect of *Terminalia arjuna* bark powder on some diagnostic enzymes in buffalo (*Bubalus bubalis)* ingesting arsenic contaminated water and fodder

**DOI:** 10.14202/vetworld.2016.1167-1172

**Published:** 2016-10-31

**Authors:** Subrat Kumar Dash, Shashi Nayyar, Rajesh Jindal

**Affiliations:** Department of Veterinary Physiology and Biochemistry, College of Veterinary Science, Guru Angad Dev Veterinary and Animal Sciences University, Ludhiana - 141 004, Punjab, India

**Keywords:** arsenic, *Bubalus bubalis*, diagnostic enzymes, *Terminalia arjuna*

## Abstract

**Aim:**

The study investigated the effect of *Terminalia arjuna* bark powder on some diagnostic enzymes related to hepatic and muscle function in buffaloes ingesting arsenic contaminated water and fodder in an arsenic affected area.

**Materials and Methods:**

A total of 25 samples of tube well water, fodder and buffalo blood were collected through a survey from arsenic contaminated areas and 20 samples from the uncontaminated, i.e., control areas of Ludhiana district, Punjab for determination of arsenic concentration. A total of 30 buffaloes (selected from above 45 animals) were divided into three groups of 10 each on the basis of blood arsenic level, *viz*., control group: Clinically healthy buffaloes from the uncontaminated area with the blood arsenic level within the normal limit (0-0.05 ppm); Arsenic exposed group: Buffaloes exposed to arsenic through intake of contaminated water and fodder in the arsenic affected area with the blood arsenic level above the normal limit of 0-0.05 ppm; treatment group: Arsenic exposed buffaloes treated with *T. arjuna* bark powder orally at 42 mg/kg b.w. OD for 30 days. Single blood samples were collected from control and arsenic exposed groups. Blood samples from the treatment group were collected on 0, 15^th^, and 30^th^ day of treatment along with one sample on the 45^th^ day, i.e., after withdrawal of treatment. Activities of alkaline phosphatase (ALP), gamma-glutamyl transferase (GGT), lactate dehydrogenase (LDH), and creatine kinase (CK) were assayed in plasma.

**Results:**

Significantly (p<0.05) higher arsenic concentration was observed in tube well water, fodder and buffalo blood samples collected from the arsenic contaminated area. A significant positive correlation was noticed between arsenic concentrations of tube well water, fodder and untreated buffalo blood samples, collected from the arsenic affected area. ALP, GGT, LDH, and CK activities were significantly (p<0.05) increased in the arsenic exposed buffaloes compared to control. Treatment with *T. arjuna* bark powder reduced the plasma levels of ALP, GGT, LDH, and CK in arsenic exposed buffaloes comparable to that of control.

**Conclusion:**

Exposure to arsenic altered the hepatic and muscle function enzymes in buffaloes. *T. arjuna* bark powder supplementation lowered the ALP, GGT, LDH, and CK activities in arsenic exposed buffaloes toward the values exhibited by the control group.

## Introduction

Arsenic, a naturally occurring metalloid is present throughout the ecosystem and biological systems [1]. Arsenic has been detected in fodder, crops and water in different concentrations in many geographical locations including different regions of Punjab, India [2]. Contamination of drinking water through natural release of arsenic from aquifer rocks is the primary source of exposure in farm animals [1]. Apart from drinking water, livestock fed with green fodder grown in arsenic-contaminated area is another source of exposure to arsenic [3]. Arsenic can cause several health problems in animals and human [4]. Exposure to arsenic can affect the function of liver, kidney, heart, and brain in cattle and mice [5,6] which were supported by altered biochemical profile [1]. Arsenic undergoes biotransformation reaction in liver and can cause hepatic injury [7]. Altered muscle function due to arsenic exposure has also been documented [7]. Injury to both liver and muscle releases cellular enzymes to blood that can be used as useful parameters for determining the hepatic and muscle function. Traditionally, ethnomedicines are extensively used in India for the treatment of various disorders due to their easy accessibility, low cost, and fewer side effects [8]. In recent years, there is increasing demand of plant-derived therapeutics. Medicinal herbs have proven hepatoprotective and myoprotective potential [9]. Powders and extracts prepared from them are widely used in the treatment of liver diseases such as hepatitis, cirrhosis, and loss of appetite [9].

The bark of *Terminalia arjuna*, a deciduous tree of the Combretaceae family, has been reported in ancient Indian medicinal literature as well as in current literatures for having beneficial effects on various chemical mediated disorders [5]. *T. arjuna* bark contains many active constituents such as tannins, triterpenoid saponins (arjunic acid, arjunolic acid, arjungenin, and arjunglycosides), flavonoids, ellagic acid, gallic acid, oligomeric proanthocyanidins, phytosterols, calcium, magnesium, zinc, copper, and coenzyme-Q [9]. Hepatoprotective, myoprotective, antioxidant and anti-inflammatory activity of *T. arjuna* dried bark powder have been studied using laboratory animal model [10]. This study was planned to evaluate the effect of *T. arjuna* dried bark powder on hepatic and muscle function enzymes in buffaloes reared in arsenic contaminated area of Ludhiana district, Punjab, India.

## Materials and Methods

### Ethical approval

Experimental protocols using buffaloes in this study have been approved by the Institutional Animal Ethical Committee (IAEC) of the Guru Angad Dev Veterinary and Animal Sciences University, Ludhiana, Punjab, India. All the experiments with buffalo were carried out according to the guidelines of the IAEC.

### Location of study and animals

An arsenic affected area was selected through a survey in Ludhiana district, Punjab, India, where the drinking water arsenic concentration was above the maximum permissible limit (i.e., 0.01 ppm) described by WHO (2005). Another area 40 km distant from contaminated site without any arsenic contamination problem was taken as control area. Water, fodder and buffalo blood samples were collected both from the arsenic contaminated and uncontaminated areas. The experiment using adult female Murrah buffaloes (3-5 years of age) was carried out in dairy farms located in Ludhiana district, Punjab. The buffaloes were maintained by their owners in organized dairy farms and provided with standard diet and *ad libitum* water.

### Experimental procedure

#### Collection of samples

Drinking water

Tube well water samples (100 ml) were collected in duplicate both from arsenic contaminated (n=25) and control area (n=20) of Ludhiana district, Punjab. These tube wells were used for irrigation and supply of drinking water to the buffaloes. Water samples were collected in polypropylene bottles prewashed with nitric acid (1 ml/L) [11]. Collected water samples were preserved in concentrated hydrochloric acid (4 ml/L) and stored at 4°C in refrigerator till estimation of total arsenic.

Fodder

Fodder samples used for feeding of buffaloes were collected in duplicate both from arsenic (n=25) contaminated and control area (n=20). These samples were washed with 2% hydrochloric acid and distilled water to remove all the impurities and dust particles [2]. After removing the extra water with blotting paper, samples were cut into pieces, packed into Petri dishes, and kept in an oven for drying. The dried samples were grinded and passed through a sieve of 2 mm size and then kept at room temperature till estimation of arsenic.

Blood

Jugular vein blood (3 ml) was collected from the buffaloes both from the arsenic contaminated (n=25) and control area (n=20) using a sterile syringe and needle and transferred to a heparinized vial. Blood was transported from the field in an ice box. The whole blood samples were used for quantitation of total arsenic.

### Determination of arsenic concentration

#### Glassware decontamination

All glassware used for estimation of arsenic were washed in detergent, soaked overnight in chromic acid and rinsed several times with distilled water before drying in hot air oven.

#### Processing of samples

Water

Water samples were directly analyzed for the estimation of total arsenic [12].

Fodder

Fodder samples (1 g) were digested using 15 ml of tri-acid mixture (HNO_3_, H_2_SO_4_, and HClO_4_ in 10:4:1 ratio) until a transparent solution was obtained [13]. After cooling, the digested sample was filtered using Whatman No. 42 filter paper, and the volume was made to 10 ml with doubled distilled water.

Blood

Blood samples (3 ml) were digested after adding 15 ml of tri-acid mixture (HNO_3_, H_2_SO_4_, and HClO_4_ in 10:4:1 ratio) until a transparent solution was obtained [14]. After cooling, the digested samples were filtered using Whatman No. 42 filter paper and the volume was made to 10 ml with doubled distilled water.

### Estimation of arsenic

The concentration of arsenic in the water, fodder and blood samples was estimated using atomic absorption spectrophotometer (AAnalyst 700, Perkin Elmer, Germany) with a flow injection hydride generation system (FIAS 100). All the determinations were performed in duplicate.

### Procurement of *T. arjuna* bark powder

*T. arjuna* bark powder used for the treatment of buffaloes was obtained from Nature Natural Ayurvedic Life Care (Bhubaneswar, Odisha, India).

### Quality criteria and selection of dose

The quality of *T. arjuna* bark powder was maintained by the Ayurvedic Medicine Company which was congruent to the Ayurvedic Pharmacopoeia of India (API), Department of Health and Family Welfare, Government of India as well as to Indian Pharmacopoeia (Indian Pharmacopoeia Commission 2010). The dose of *T. arjuna* bark powder (42 mg/kg b.w.) was decided as per the API, Department of Health and Family Welfare, Government of India.

### Grouping of buffaloes

A total of 30 adult female Murrah buffaloes (out of 45 animals selected through survey for determination of blood arsenic level) were divided into following three groups:

#### Control group (n=10)

Clinically healthy buffaloes selected from the uncontaminated area without any treatment (with blood arsenic level within the normal limit [0-0.05 ppm]).

#### Arsenic exposed/exposure control group (n=10)

Buffaloes exposed to arsenic through intake of contaminated water and fodder selected from the arsenic contaminated area (with blood arsenic level above the normal limit of 0-0.05 ppm [1]).

#### Treatment group (n=10)

Arsenic exposed buffaloes treated with *T. arjuna* bark powder at 42 mg/kg b.w. for 30 days.

All the buffaloes were of same age and body weight (approximately). The treatment schedule did not cause any change in feed and water intake pattern of animals. Three blood samples (3 ml) were collected during the supplementation period, i.e., on 0, 15^th^, and 30^th^ day and one blood sample was collected after the withdrawal of supplementation, i.e., on 45^th^ day from each buffalo of the treatment group. Single blood samples were collected from each animal of the control and arsenic exposed groups. The blood samples were centrifuged at 2500 rpm for 10 min for separation of plasma.

### Biochemical analysis

Liver function parameters, *viz*., alkaline phosphatase (ALP) and gamma-glutamyl transferase (GGT) and muscle function indices, *viz*., lactate dehydrogenase (LDH) and creatine kinase (CK) were determined in plasma using Autopak kits (Siemens Healthcare Diagnostics Ltd.) on semi-automatic biochemical analyzer. All the determinations were performed in duplicate.

### Statistical analysis

Data obtained were analyzed with Statistical Package for Social Sciences software (Version 16.0). The results were expressed as mean±SE of mean. Multiple comparisons of data were carried out using one-way ANOVA, and the group means were compared by Duncan’s multiple range test. Additional statistical comparisons between means of different groups were carried out using independent t-tests. Correlation between different parameters was determined by Karl Pearson’s correlation coefficient.

## Results

**Figure-1:**
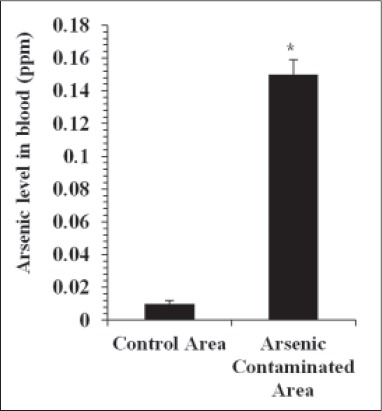
Totalarsenic level (mean±SE) in buffalo blood samples collected from control (n=20) and arsenic contaminated (n=25) area. *Indicates significant difference at p<0.05. Note: Normal limit of arsenic in bovine blood is 0-0.05 ppm [1].

In this study, arsenic concentration in water and fodder samples (Table-1) was reported to be significantly (p<0.05) increased in the exposed area of Ludhiana district compared to the control area. The mean arsenic level in water and fodder was observed to be 12.5-fold and 34-fold higher, respectively, in the contaminated area compared to control. However, the arsenic level in fodder was within the maximum permissible limit for fodder [2]. Blood samples collected from the buffaloes of arsenic exposed area showed significantly (p<0.05) elevated arsenic concentration compared to control area (Figure-1). Significant positive relationship (r=0.516, p<0.05) was observed between arsenic concentration in tube well water and fodder in the exposed area. Tube well water arsenic level showed close positive relationship (r=0.821, p<0.05) with whole blood arsenic level in the exposed area before *T. arjuna* bark powder treatment. Arsenic level in fodder samples was significantly correlated with the arsenic concentration in untreated buffalo blood samples collected from the contaminated area (Table-2). Activities of liver and muscle function enzymes in buffaloes were observed to be affected by the arsenic exposure. The ALP, GGT, CK, and LDH activities were significantly (p<0.05) elevated in the arsenic exposed buffaloes with 1.84-, 1.70-, 1.90-, and 1.45-fold increase, respectively (Table-3). Treatment with *T. arjuna* bark powder at 42 mg/kg b.w. for 15 days did not affect the ALP activity of treatment group compared to control (p<0.05). However, the treatment in the same dosage for 30 days significantly declined the ALP activity comparable to that of control group which was remained unchanged after withdrawal of supplementation. The treatment with *T. arjuna* bark powder for 15 days significantly decreased the GGT, CK, and LDH activities in the treatment group compared to control which remained stable after withdrawal of treatment as observed at day 45.

**Table-1:** Arsenic concentration (mean±SE) in tube well water and fodder samples from control and arsenic contaminated area.

Samples	Arsenic concentration (ppm)	

	Control area (n=20)	Arsenic contaminated area (n=25)
Tube well water	0.004±0.001	0.05±0.01*
Fodder	0.01±0.004	0.34±0.03*

* Indicates significant difference at P<0.05. Note: Maximum permissible limit of arsenic in drinking water in view of animal health is 0.01 ppm [15]. Phytotoxicity limit of arsenic is 1 ppm dry weight [2]

**Table-2:** Relationship between arsenic concentration in tube well water, fodder and untreated buffalo blood samples of arsenic contaminated area.

Parameters	Pearson’s correlation coefficient (r)
Arsenic_(Tube well water)_ versus arsenic_(Fodder)_	0.516*
Arsenic_(Tube well water)_ versus arsenic_(Buffalo blood)_	0.821*
Arsenic_(Fodder)_ versus arsenic_(Buffalo blood)_	0.672*

* Indicates significant difference at P<0.05; Number of each type of sample=25

**Table-3:** Liver and muscle function enzymes (U/L) (mean±SE) in control, arsenic exposed and *Terminalia arjuna* bark powder treated buffaloes.

Parameters	Control group (n=10)	Arsenic exposed group (n=10)	Treatment group (n=10)			

			During treatment			After withdrawal of treatment
	
			0 day	15^th^ day	30^th^ day	45^th^ day
ALP	152.41±1.51^a^	281.56±2.05^b^	283.12±1.93^b^	174.61±2.13^c^	155.12±1.43^a^	153.41±1.17^a^
GGT	15.52±0.73^a^	26.41±1.21^b^	25.63±1.21^b^	18.02±1.21^a^	16.71±1.21^a^	16.84±1.21^a^
CK	6.17±0.32^a^	11.74±0.11^b^	12.15±0.18^b^	9.03±0.83^a^	7.51±0.43^a^	6.26±0.41^a^
LDH	786.4±2.18^a^	1143.16±2.51^b^	1147.41±1.85^b^	785.25±2.03^a^	789.25±1.75^a^	787.16±1.26^a^

Within each row, means with different superscripts (a, b, c) are significantly different (p<0.05)

## Discussion

Arsenic contamination through the environment in different parts of the world is a matter of concern [16]. Large numbers of animals especially of India and Bangladesh are reported to be affected with arsenic toxicity through drinking water and consumption of contaminated fodder [1]. Ludhiana, the industrial hub of Punjab is currently at risk of arsenic toxicity [2]. In Punjab, dairy animals are mostly supplied with the tube well water for drinking. More use of underground water has lowered the water level that carries arsenic-containing salts and minerals. High pH (>8.0) and high concentrations of phosphate, sulfate, borate, and hydroxyl anions in the ground water aquifers of Punjab are responsible for the widespread release of arsenic concentrations from sulfide oxidation and oxyhydroxide of iron [2]. Hence, greater level of arsenic in tube well water samples probably indicates that source of arsenic is natural rather than anthropogenic. In Punjab, the dairy animals are mainly maintained in organized dairy farms and fed with chopped fodders. Arsenic concentration in fodder was reported to be higher in exposed area but was well below the maximum permissible limit for fodders [2]. Fodders seldom accumulate arsenic at concentrations hazardous to human and animal health because phytotoxicity usually occurs much before such threshold concentrations are reached in plants [17]. Increased arsenic level in fodder reported in this study could be a threat to the buffaloes because they are mainly fed with the green fodder in Punjab. Higher arsenic level observed in blood of exposed buffaloes in this study could be a result of intake of arsenic mainly through contaminated drinking water and fodder. The close relationship between water, fodder and buffalo blood samples further support the fact that elevated blood arsenic level in buffaloes is probably due to the intake of contaminated water and fodder that might predispose the buffaloes to the risk of development of sub-clinical toxicity. It is reported that most toxic elements undergo biotransformation in the liver and cause hepatic as well as systemic injury [1]. Arsenic-mediated oxidative stress was also reported to have damaging effects on hepatocytes and myocytes releasing the related enzymes to the bloodstream [7]. Increased ALP and GGT activity observed in the buffaloes of arsenic contaminated area in this study might be resulted from arsenic-mediated hepatic injury. Different pharmaceutical and toxic agents can cause hepatotoxicity resulting in the increased serum ALP activity in domestic animals [18]. In liver, GGT is primarily associated with biliary epithelial cells and reversible hepatic injury induced by different toxic chemicals results in minimal change in plasma GGT activity [9]. Increased serum GGT activity was also reported in various cases of plant and chemical related hepatotoxicity in cattle [9] which is in line with the results of this study. Various toxic agents including metals can cause myonecrosis that resulted in elevated plasma CK activity [19] which is in accordance with this study. LDH is a non-specific enzyme and elevated plasma activities have been observed in hepatic as well as myonecrosis [18]. Higher plasma LDH level in cattle has been associated with metal toxicity [19]. Increased plasma LDH level in buffaloes as observed in this study was in line with previous studies. Restoration of plasma ALP, GGT, CK, and LDH activity toward normal control values in arsenic exposed buffaloes after administration of *T. arjuna* bark powder observed in this study confirmed its hepatoprotective and myoprotective activity. Elevated levels of plasma ALP and GGT are indicative of cellular leakage and loss of functional integrity of cell membrane in liver cells [9]. In this study, the ALP and GGT levels returned to normal after treatment with *T. arjuna* bark powder which might be due to healing of parenchyma and regeneration of hepatocytes [9]. The previous study on *T. arjuna* bark extract was reported to decline the elevated level of ALP and GGT in isoniazid and paracetamol included hepatotoxicity [9]. The hepatoprotective activity might be due to the presence of high content of phenolic derivatives in *T. arjuna* bark powder [9]. *T. arjuna* has been known as a potential myoprotective and cardioprotective agent since vedic period [6]. *T. arjuna* bark powder improves the muscle tone and strength. It is rich in coenzyme-Q which is required by muscles for its energy requirements [9] and has therapeutic effect against muscle cell damage and myonecrosis [6]. The restoration of LDH and CK activities in arsenic exposed buffalo treated with *T. arjuna* bark powder might be due to its protective effect on muscle cells.

## Conclusion

Natural exposure to arsenic contaminated water and fodder altered the hepatic and muscle function enzymes in buffaloes. Treatment with *T. arjuna* bark powder succeeded to decline the ALP, GGT, LDH, and CK activities in arsenic exposed buffaloes toward the normal control values. The bark of this plant could be very useful as a hepatoprotectant and muscle protectant. However, further studies are required to identify the exact phytochemical responsible for these curative effects.

## Authors’ Contributions

SN, RJ, and SKD designed the experiment supervised by SN. With the help of RJ and SN, SKD collected the samples and performed the laboratory analysis. SKD along with SN analyzed the data and prepared the manuscript. SN, RJ, and SKD reviewed the manuscript. All authors read and approved the final manuscript.
